# The Distribution and Localization of Collagen Triple Helix Repeat Containing-1 in Naturally and Experimentally Avian Leukosis Virus Subgroup J-Infected Chickens

**DOI:** 10.3389/fvets.2020.565773

**Published:** 2020-09-25

**Authors:** Yu Pang, Defang Zhou, Jing Zhou, Jingwen Xue, Yiya Wang, Ziqiang Cheng

**Affiliations:** ^1^College of Veterinary Medicine, Shandong Agricultural University, Tai'an, China; ^2^School of Life Sciences, Qilu Normal University, Jinan, China

**Keywords:** ALV-J, Cthrc1, IHC, Myelocytomas, Poultry

## Abstract

Collagen triple helix repeat containing-1 (CTHRC1) has recently been identified as avian leukosis virus subgroup J (ALV-J) replication-dependent factor that remarkably facilitates ALV-J replication via interaction with the envelope glycoprotein (SU) of ALV-J. However, the dynamic distribution and localization of CTHRC1 in various tissues upon ALV-J infection are still unknown. In this study, data revealed that the levels of CTHRC1 were significantly increased in various tissues and that the protein was mainly located in the cytoplasm and nucleus of parenchymal cells in tissues of chickens that were infected by ALV-J naturally and experimentally. Interestingly, CTHRC1 was also observed in leukocytes other than erythrocytes in congested veins of ALV-J-infected tissues. Consequently, the positive cells in these veins were confirmed as lymphocytes by laser confocal microscopy. Taken together, these results conclude that the CTHRC1 is an inducible protein and exhibited ubiquitous expression in ALV-J-infected chickens, which may provide basic information for in-depth study of ALV-J infection and replication mechanisms.

## Introduction

Avian leukosis virus subgroups (ALVs) belonging to type C *retroviruses* are generally divided into seven subgroups (A–E, J, and K) (1–3). It is noteworthy that since isolation of the first strain (HPRS-103) from meat-type chickens in the United Kingdom in the late 1980s, more attention has been focused on the J subgroup of ALV (ALV-J), which caused high mortality, wide host ranges, and strong tumorigenicity (4, 5). Thus, it is absolutely imperative that the strategies should be implemented to prevent and control ALV-J infection in poultry industry. Attributed to the implementation of ALV-J eradication program (6), there were rarely reports of ALV-J or myelocytomatosis in China after 2013. Nevertheless, there were another intensive reports about ALV-J breakout almost simultaneously in six provinces of China in 2018, which attracted our attention (7). It is rather remarkable that there were extensive medullary-like tumor cells in the bone marrow, liver, and kidney, and all of the neoplasms were myelocytomas (7).

In recent years, a variety of studies have explored that collagen triple helix repeat containing-1 (CTHRC1) highly related to collagen synthesis was remarkably up-regulated in solid tumors (8–10), including hepatocellular carcinoma (11, 12), oral squamous cell carcinoma (13, 14), gastric carcinogenesis (9, 15), ductal carcinoma (16), and colorectal cancer (17–19), and that the expression abundance of CTHRC1 was significant low in normal tissues. CTHRC1 is a highly conserved cancer-secreted glycosylated protein; human CTHRC1 shares 80% sequence identity with the *Gallus* homolog, which was discovered in a screening for differentially expressed genes in a rat model of balloon-injured vasculature (15, 20–22). Thus far, the studies of CTHRC1 focus on the field of oncology; there is rare coverage on the role of CTHRC1 in the field of viral replication. Recently, we have firstly reported that CTHRC1 facilitates ALV-J replication through interaction with the SU protein of ALV-J, which indicates that CTHRC1 plays a crucial role in regulating ALV-J replication (23). These studies may provide new enlightenment to the study of interaction between host protein(s) and ALV-J, and these expand the biological function of CTHRC1 in the field of virus replication (23). However, the distribution and expression of CTHRC1 in chicken tissues upon ALV-J infection are still unknown, which may provide a theoretical basis for the subsequent research of ALV-J and CTHRC1.

In the study, we investigated for the first time the expression and distribution of CTHRC1 in chickens that were infected with ALV-J naturally and experimentally. Interestingly, we found that ALV-J could infect with lymphocytes and activate the expression of CTHRC1 in the lymphocytes.

## Materials and Methods

### Virus, Antibodies, and Experimental Animals

ALV-J strain GM0209 was isolated from a broiler breeder maintained in our laboratory (7). Monoclonal antibody directed against ALV-J SU protein was produced as described previously (23, 24). Rabbit polyclonal antibody against CTHRC1 was purchased from Absin Corporation (Shanghai, China). Specific pathogen free (SPF) chick embryos were acquired from the SPAFAS Corporation (Jinan, China; a joint venture with Charles River Lab, Wilmington, MA, USA). Chick embryos were incubated in an SPF environment at the Laboratory Animal and Resources Facility, Shandong Agricultural University. Chick embryos were inoculated with ALV-J (GM0209 strain) via allantoic cavity at the sixth embryonic age, with the titer of 10^3.8^ tissue culture infective dose (TCID_50_)/0.1 ml. Chicks then were euthanized at 15, 22, and 35 days post-infection. Chick heart, liver, kidney, spleen, lung, brain, duodenum, proventriculus, and bone marrow (thymus and bursa of Fabricius will not be discussed in this article) were collected and fixed in 4% formaldehyde solution. Animal experimental protocols were approved by the Shandong Agricultural University Animal Care and Use Committee (permit No. SDAU 19-098; July 8, 2019).

### Tissue Information of Natural Avian Leukosis Virus Subgroup J-Infected Chickens

ALV-J infection broke out in Jiangsu, Shandong, Henan, Hebei, Heilongjiang, and Guangdong provinces of China in February 2018 (7). On-site, a total of 19,500 Ross broiler breeder chickens aged 15–20 weeks suffered from depression, paralysis, and weight loss. Tissues samples including the liver, kidney, and bone marrow were collected from broiler breeder flocks in Gaomi and Binzhou. Samples were verified that the case was only ALV-J infection by performing hematoxylin and eosin (HE) staining and PCR (7).

### Immunohistochemistry Staining

The tissue microarray glass slides were prepared using the tissues of naturally and experimentally ALV-J-infected chickens and normal chickens. Then, the tissue microarray glass slides were dried at 60°C for 30 min and then deparaffinized gradually through xylene, 50% xylene, and gradient concentrations of ethanol until being immersed in water (25). Chicken tissue sections were blocked for peroxidase activity with 0.3% hydrogen peroxide for 40 min. Antigen retrieval was performed through boiling in 10 mmol/L citrate buffer (pH 6.0) for 25 min. Then the tissues were incubated with anti-CTHRC1 antibody (1:100 dilution) at 4°C for 12 h. The tissues were washed with phosphate-buffered saline (PBS) for three times and incubated with horseradish peroxidase (HRP)-labeled anti-rabbit secondary antibody (1:500 dilution) for 70 min at 37°C. Immunostaining was performed by using diaminobenzidine substrate chromogen method. Tissues were immersed in hematoxylin for cell nuclear staining. The slides were then dehydrated via gradient concentrations of ethanol, cleared with xylene, and cover slipped with neutral balsam (Shenggong, Shanghai, China) (26).

### Immunofluorescence Staining

Lymphocytes and erythrocytes were separated from the peripheral blood of ALV-J-infected and normal chickens. Lymphocytes and erythrocytes were fixed with 4% paraformaldehyde and then permeabilized with methanol for 20 min prior to the addition of primary antibody (anti-mouse ALV-J SU protein and anti-rabbit CTHRC1) and incubation at 37°C for 60 min. These two types of cells then were washed in Tris-buffered saline-Tween 20, followed by incubation with Alexa Fluor 488-conjugated donkey anti-rabbit or 594-conjugated donkey anti-mouse antibodies. Cell nuclei were stained with 4′,6-diamidino-2-phenylindole (DAPI). Finally, lymphocytes and erythrocytes were mixed in a 1:1 ratio. Fluorescent images were obtained with a confocal laser scanning microscope (Leica SP8).

### Reverse Transcription and Quantitative Real-Time PCR and PCR

Total RNA was extracted from cells according to manufacturer instructions (Qiagen), and RNA concentration was measured using a spectrophotometer (Qiagen). We used 1 μg of total RNA as a template to synthesize cDNA using a reverse transcriptase kit (TaKaRa, Shiga, Japan) according to manufacturer instructions. A SYBR Green I kit (TaKaRa) was used for cDNA amplification in a total volume of 20 μl. CTHRC1-specific primers and glyceraldehyde 3-phosphate dehydrogenase (GAPDH), a housekeeping gene, are shown in Table 1. A LightCycler 96 system (Roche, Basel, Switzerland) was used for qPCR with the following cycling conditions: denaturation at 95°C for 30 s, followed by 40 cycles at 95°C for 5 s, and 60°C for 34 s. A melting curve was generated at 95°C for 10 s, 65°C for 60 s, and 97°C for 1 s. CTHRC1 levels were analyzed using the 2^−ΔΔCt^ method. Total RNA was extracted from clinical case, and then RNA is reverse transcribed into cDNA. PCR was performed according to the manufacturer's instructions of PrimeSTAR HS DNA Polymerase with GC Buffer (Takara, Japan) (PCR primer is shown in Table 1).

**Table 1:** Primer used for real-time PCR.

**Name**	**Sequence (5^**′**^-3^**′**^)**	**Reference or GenBank**
GAPDH	F: GAACATCATCCCAGCGTCCA	(23)
	R: CGGCAGGTCAGGTCAACAAC	
CTHRC1	F: ACGCTG GCTTGGTGGA	(23)
	R: CAGTTCTTCAATGATGATACGG	
ALV-A	F: GATGTTCACTTACTCGAGC	GenBank: MF926337.1
	R: CGTTTACGTCTTATACCTG	
ALV-B	F: ATGTCCACTTACTCGAGCA	GenBank: KC282901.1
	R: TCGTTTGCGTCTTATACCTG	
ALV-J	F: AACAGGTTACATCTGAGCAAGC	GenBank: DQ115805.1
	R: TGTTCCATTGTCATCGCTAACG	
ALV-K	F: CTCGAGCAGCCAGGGAAC	GenBank: KY581580.1
	R: CTTCGTTTACGTCTTATACC	
MDV	F: GCCTTTTATACACAAGAGCCGAG	(27)
	R: TTTATCGCGGTTGTGGGTCATG′	
REV	F: CATACTGGAGCCAATGGTT	(28)
	R: AATGTTGTAGCGAAGTACT	

### ELISA

Virus isolated from clinical cases was inoculated into DF-1 cells, where are a continuous chicken embryonic fibroblast cell line that exhibits normal fibroblastic morphology and is free of endogenous sequences related to leukosis viruses and avian sarcoma (29). Importantly, DF-1 cells are capable of sustaining ALV-J replication, and the cell supernatant was collected at different time points. ELISA kits (SenBeiJia, Nanjing, China) (http://www.sbjbio.com/Products/P1.html) were used to measure CTHRC1 levels in cell supernatant, according to manufacturer instructions. Each experiment included three biological replicates.

### Statistical Analysis

All data are reported as the mean ± standard error of the mean (SEM). Prism 7.0 software (GraphPad Software, San Diego, CA, USA) was used to determine statistically significant differences by performing a two-tailed unpaired Student's *t*-test. Differences between groups were considered statistically significant when the *p*-value was less than 0.05 (^*^p < 0.05; ^**^p < 0.01; ^***^p < 0.001).

## Results

### The Expression of Collagen Triple Helix Repeat Containing-1 Is Increased in Naturally and Experimentally Avian Leukosis Virus Subgroup J-Infected Chickens

Data obtained from previous studies of our lab indicated that the chickens from farms were only infected with ALV-J (7). According to Zhou et al. (7), the virus was isolated from the samples of kidneys of tumor-bearing chickens from farms (29). In order to detect the purity of the virus that we isolated, PCR was performed. The results of PCR indicated that all tumor-bearing chickens forming a clinical case were positive for ALV-J and negative for ALV-A, ALV-B, ALV-K, REV, and MDV (Supplementary Figures 1A–F) (7).

**Figure 1:**
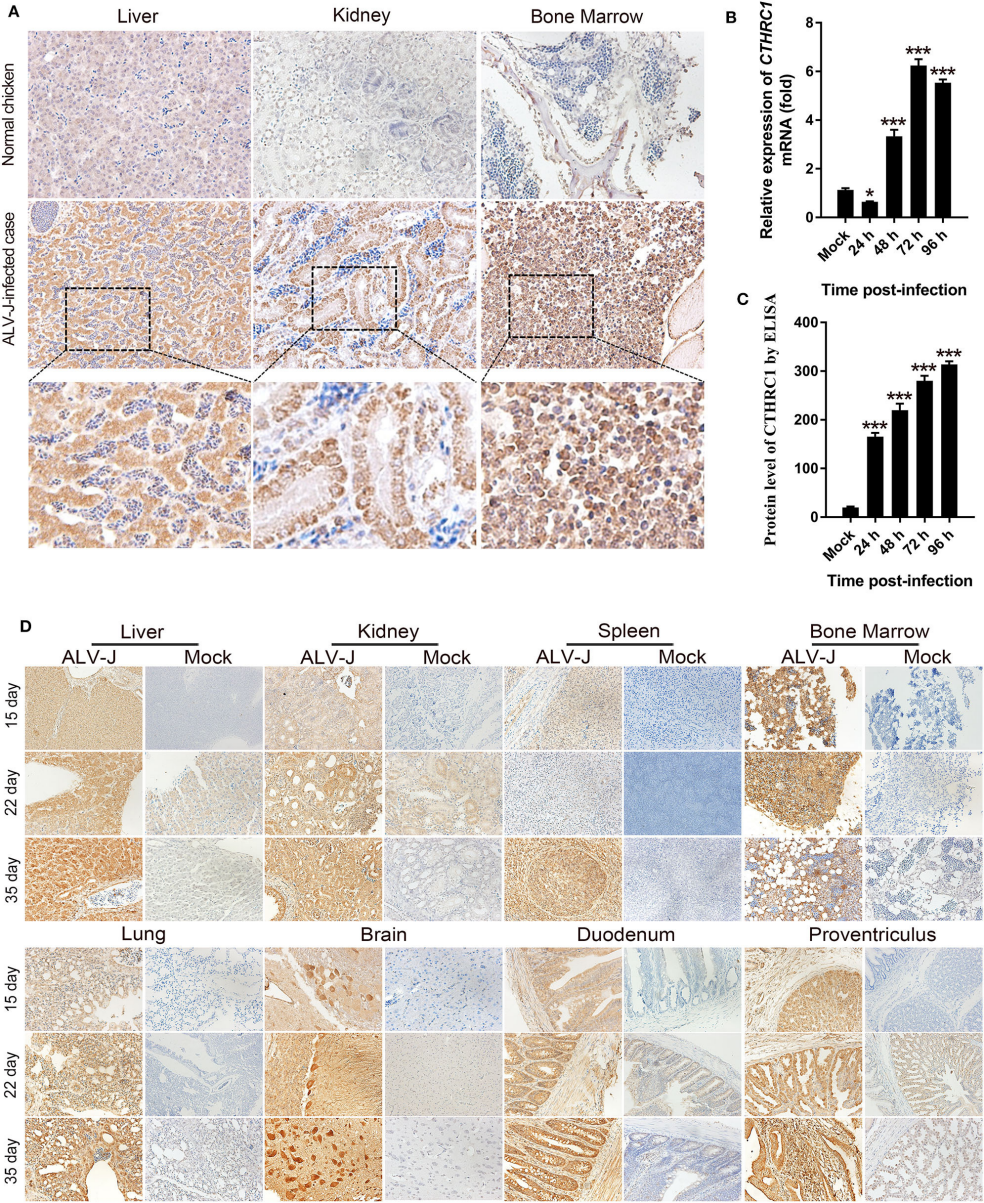
Avian leukosis virus subgroup J (ALV-J) infection facilitates collagen triple helix repeat containing-1 (CTHRC1) expression *in vivo*. Immunohistochemical staining of tissues of naturally **(A)** and experimentally **(D)** ALV-J-infected chickens with the CTHRC1 antibody. The CTHRC1 protein was weakly stained in normal tissues. In contrast, CTHRC1 staining was moderate to strong in ALV-J-infected tissues. Original magnification, 400×. **(B)** Total RNA was extracted from ALV-J-infected and uninfected DF-1 cells. CTHRC1 mRNA was measured by qPCR. **(C)** CTHRC1 protein levels were measured by ELISA in supernatant from ALV-J infected and uninfected DF-1 cells. *p < 0.05; ***p < 0.001.

The expression of CTHRC1 is low in normal tissues, which can be activated with the stimulation of certain pathogens (30, 31). Then, we detected the expression of CTHRC1 in the liver, kidney, and bone marrow of naturally ALV-J-infected chickens. As shown in Figure 1A, the levels of CTHRC1 were remarkably up-regulated in the tissues of naturally ALV-J-infected chickens, and the expression of CTHRC1 was mainly and widely distributed with the parenchymal parts of organs, compared with the normal tissues of the same age of SPF chickens. CTHRC1 is widely expressed in the tissues of clinical cases infected with ALV-J. In order to further clarify that CTHRC1 is an inducible protein upon ALV-J infection, we inoculated the virus of isolated into DF-1 cells and then tested whether ALV-J up-regulated the expression of CTHRC1. The results showed that CTHRC1 was elevated upon ALV-J infection (Figures 1B,C).

In order to accurately clarify the dynamic distribution of CTHRC1 in various organs upon ALV-J infection and the expression of CTHRC1 with the extension of infection time, SPF chick embryos were inoculated with ALV-J in the allantoic cavity on the 6th day of incubation, and then chicks were euthanized at 15, 22, and 35 days post-infection. As shown in Figure 1D, the levels of CTHRC1 were obviously raised upon ALV-J infection than those of normal SPF chickens. Meanwhile, we found that CTHRC1 was mainly expressed in the parenchymal cells of the tissues than those of the interstitial cells of tissues. It was worth noting that the expression of CTHRC1 was lower in the spleen than those of other organs, such as the heart, liver, kidney, lung, brain, duodenum, the proventriculus; and the expression of CTHRC1 featured high intensity in the Purkinje cells of the brain tissue.

### Collagen Triple Helix Repeat Containing-1 is Activated in Blood Lymphocytes of Avian Leukosis Virus Subgroup J-Infected Chickens

**Figure 2:**
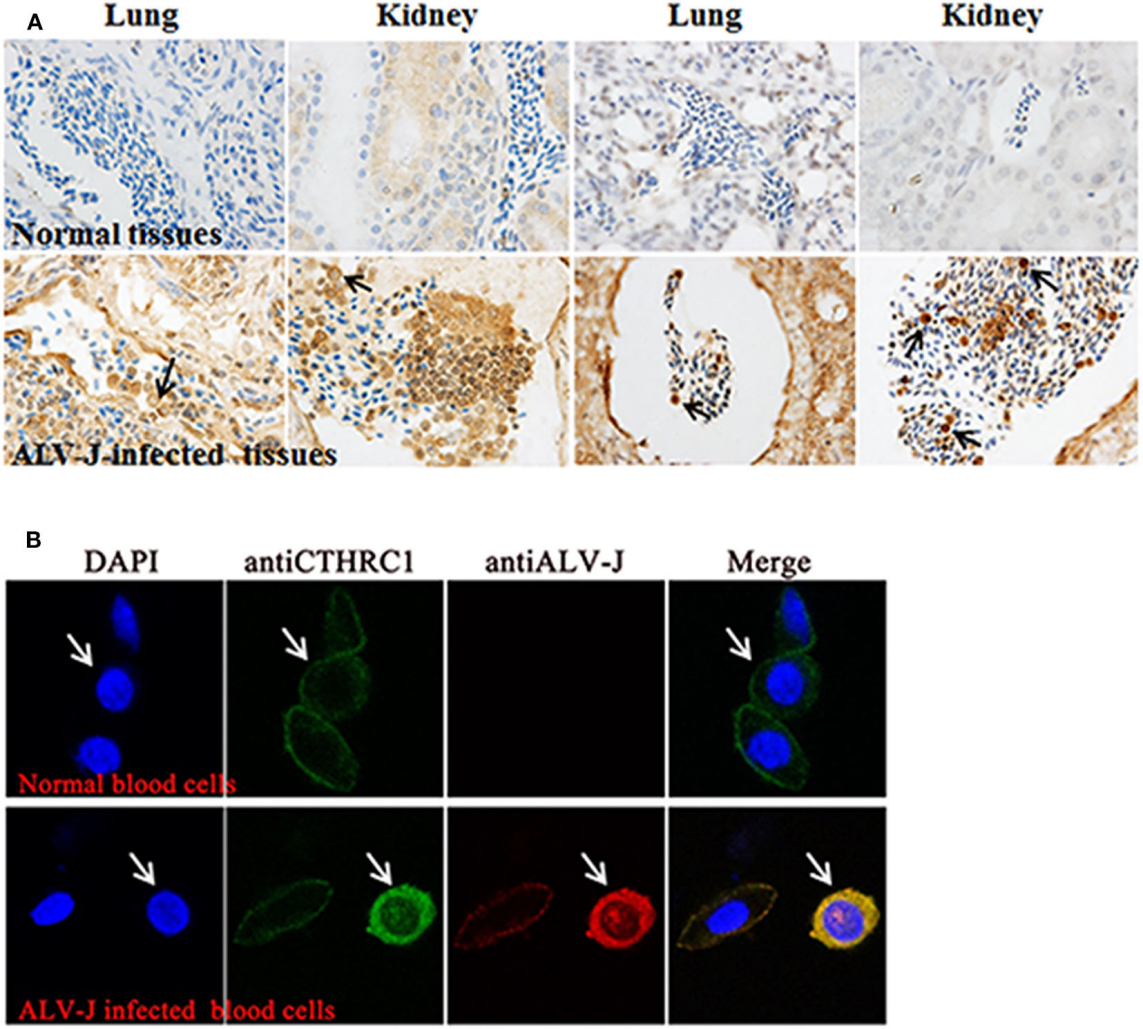
Avian leukosis virus subgroup J (ALV-J) activates the expression of collagen triple helix repeat containing-1 (CTHRC1) in blood lymphocytes **(A)**. The CTHRC1 protein was strongly stained in certain blood cells tested by the immunohistochemistry (IHC) assay. (The lungs and kidneys are derived from different ALV-J-infected chickens.) **(B)** Lymphocytes and erythrocytes isolated from the blood of ALV-J-infected chicken. Cells were fixed for an immunofluorescence assay to detect ALV-J (red) and CTHRC1 (green) with primary antibodies. Nuclei were counterstained with 4′,6-diamidino-2-phenylindole (DAPI) (blue). Fluorescent images were acquired with a confocal laser scanning microscope (Leica SP8). White arrows indicate lymphocytes.

Interestingly, during immunohistochemistry (IHC), we found that CTHRC1 was stained in certain blood cells of congested veins and that erythrocytes were not stained. Thus, we speculated that the stained cells were lymphocytes according to cell morphology (Figure 2A). To further identify the stained blood cells, lymphocytes, and erythrocytes were isolated from blood of ALV-J-infected chickens and normal chickens to exactly explore the phenomenon by laser confocal assay. As shown in Figure 2B, CTHRC1 was mainly located in the cytoplasm and was activated in the blood lymphocytes of ALV-J infected chickens than those of normal chickens. CTHRC1 was interacted with ALV-J SU protein and co-localized in the cytoplasm (23), which was in line with our results obtained in Figure 2B that CTHRC1 and ALV-J were co-located in the cytoplasm of lymphocytes. Meanwhile, according to Cui et al. (1999), ALV-J was not infected with erythrocytes of blood, which only transport ALV-J, but it can infect lymphocytes of blood (32). In our study, we found that ALV-J was only located in the cell membrane of erythrocytes (Figure 2B), which was coincident with results obtained in previous studies.

## Discussion

CTHRC1, as a host-cell protein, plays a significant role in facilitating ALV-J infection (23). Here, we explored the distribution and localization of CTHRC1 in tissues of chickens that were infected with ALV-J naturally and experimentally. Meanwhile, utilizing IHC and laser confocal assay, we found that CTHRC1 was activated in blood lymphocytes of ALV-J infection, which meant that ALV-J can infect lymphocytes.

In this study, we found that the expression of CTHRC1 gradually elevated with the extensive infection of ALV-J tested by the IHC assay. Furthermore, ALV-J-infected samples with deeper depths of invasion showed higher levels of CTHRC1, illustrating that CTHRC1 may have an irreplaceable role in ALV-J pathogenicity. It was important to note that the result was further substantiated through *in vitro* experiments (23). Therefore, CTHRC1, a candidate tumor marker, may be a potential metastasis-related gene in myelocytomas caused by ALV-J. Moreover, it was a remarkable fact that CTHRC1 staining in ALV-J-infected tissues was strongly intensely located in cytoplasm and nucleus, while the expression of CTHRC1 in normal chicken tissues was of weak intensity (Figure 1). CTHRC1 is a secreted-extracellular protein (9). Pyagay et al. (33) found that CTHRC1 may experience proteolytic process, which could acquire the activity of the molecule (31). Thus, it is possible that the secreted form of CTHRC1 overproduced through ALV-J-infection may act on the surrounding microenvironment, including the stromal cells and extracellular matrix (ECM), which elevates tumor invasion and migration. Previous studies have described that the level of CTHRC1 was very low in normal tissues, which was consistent with results obtained in Figure 1.

For young animals, the bone marrow in the marrow cavity is mainly red bone marrow, whose main function is to produce hematopoietic stem cells. The red bone marrow in the marrow cavity is gradually replaced with age by yellow bone marrow, whose main component is fat that is not stained by IHC (34). As shown in Figure 2B, after 35 days of ALV-J infection, the staining of CTHRC1 was slightly decreased, which may be because the red bone marrow in the marrow cavity is gradually replaced by the yellow bone marrow. Nowadays, effort has mainly been concentrated on the tumorigenic mechanism of ALV-J (7, 35).

As shown in Figure 1, compared with other structures of the kidney, the renal tubule is highly colored, which shows that the renal tubule highly expresses CTHRC1 after being infected with ALV-J. Enhanced expression of CTHRC1 could restrict collagen type I and III deposition (33, 36). Meanwhile, the inhibition of the expression of collagen types I and III could alter the host membrane permeability, which could promote viral replication (37). It is well-known that the kidney is the main target organ of ALV-J. It is newly revealed that the high expression of CTHRC1 in renal tubules is caused by ALV-J infection.

ALV-J belonging to an avian C type *retrovirus* can integrate into the host genome to induce tumor and immunological tolerance by impairment in the effect of host lymphocytes, which was consistent with results obtained in Figure 2B (32, 38). It can be observed that ALV-J could activate CTHRC1 in blood lymphocytes, but not in erythrocytes (Figures 2A,B), which indicated that CTHRC1 might be involved in immunological tolerance induced by ALV-J by impairing lymphocyte function. Here, CTHRC1 was found in the blood lymphocytes of ALV-J-infected SPF chickens, which revealed that it may be a potential key factor influencing ALV-J infection and production.

CTHRC1 was widely up-regulated in multiple human tumors, such as cancers of the liver, pancreas, and gastrointestinal tract and melanoma (39). Meanwhile, the recombinant of CTHRC1 protein could elevate the capacities of invasion and migration of primary gastrointestinal stromal tumors (40). Thus, CTHRC1 plays a vital role in the fields of human tumor (21). ALV-J, MDV, and REV, as the three major neoplastic diseases, cause huge economic losses to the poultry industry every year (35). However, how to prevent and control these three diseases still plague many workers. Our research found that CTHRC1 was activated upon ALV-J (23), MDV, and REV infection through proteomic analysis (data not shown), and we speculated that it may play a crucial role in ALV-J, MDV, and REV replication, tumorigenicity, and pathological mechanism and that it might also become a common target for the prevention and control of ALV-J, MDV, and REV infection. Additionally, CTHRC1 may serve as a common effective biomarker for evaluating the poor clinical pathological characteristics of ALV-J, MDV, and REV infection. In the future, we will investigate the effect of CTHRC1 on ALV-J, MDV, and REV replication, tumorigenicity, and pathological mechanism.

In conclusion, our current study revealed that CTHRC1, as an invasion-driving protein, was widely activated in the parenchymal cells of tissues of ALV-J-infected natural and experimental cases. CTHRC1 was up-regulated in blood lymphocytes, which may be associated with the immunosuppression caused by ALV-J. Thus, combined with previous studies, this research may provide precise information for in-depth study of ALV-J infection and replication mechanisms.

## Data Availability Statement

All datasets presented in this study are included in the article/Supplementary Material.

## Ethics Statement

The animal study was reviewed and approved by the Shandong Animal Care and Use Committee (permit number: SDAU number 19-098, 8 July 2019).

## Author Contributions

ZC and YP conceived and designed the experiments. YP performed the experiments. YP, DZ, JX, and JZ analyzed the data. ZC, YW, and YP wrote the paper. All authors contributed to the article and approved the submitted version.

## Conflict of Interest

The authors declare that the research was conducted in the absence of any commercial or financial relationships that could be construed as a potential conflict of interest.
