# Supplementary material for: The Distribution and Localization of Collagen Triple Helix Repeat Containing-1 in Naturally and Experimentally Avian Leukosis Virus Subgroup J-Infected Chickens
Source: Front Vet Sci. 2020 Sep 25;7:565773. doi: 10.3389/fvets.2020.565773 (PMC7546020; doi:10.3389/fvets.2020.565773)
Supplement: Supplementary file 1 [file Data_Sheet_1.docx]

Supplementary Material

1. the purity of ALV-J we isolated was detected


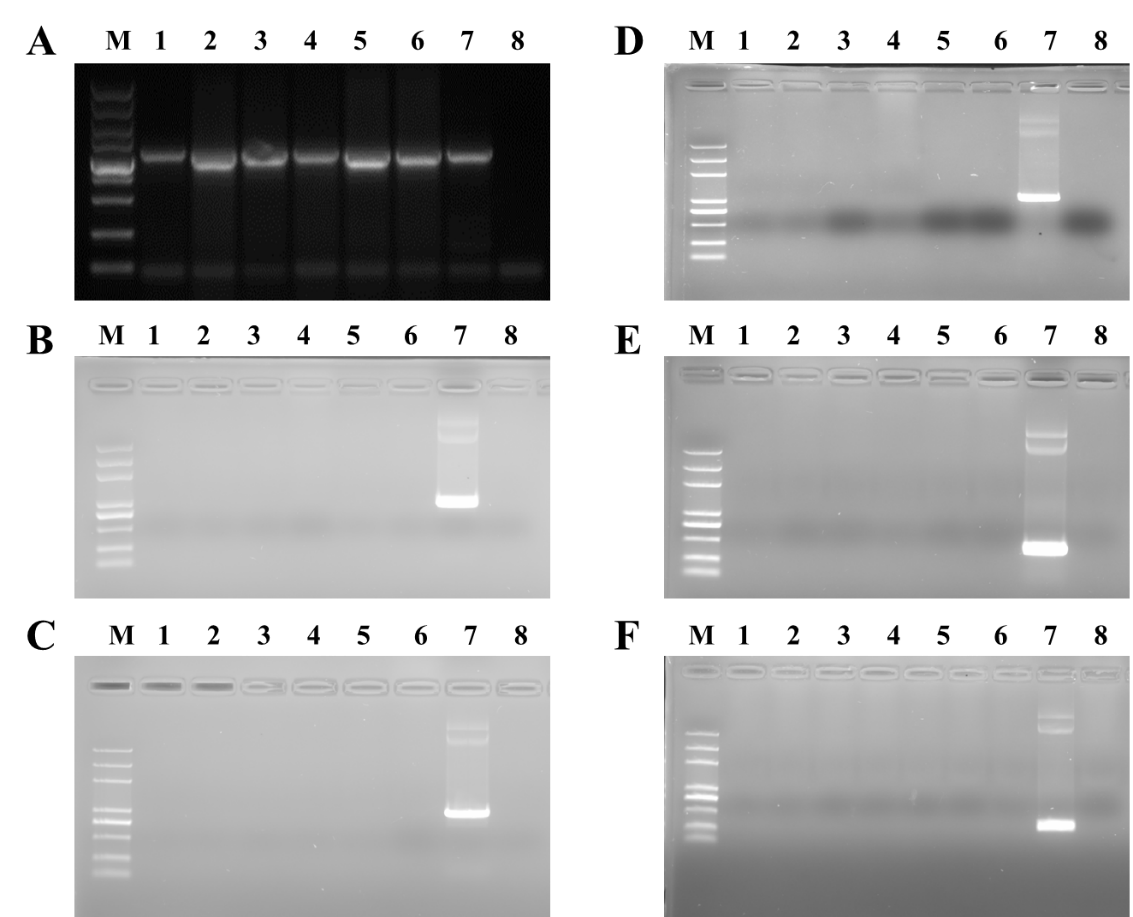


**Supplementary Figure 1.** (A) ALV-J, (B) ALV-A, (C) ALV-B, (D) ALV-K, (E) MDV and (F) REV, were detected by PCR. M: DNA Marker, DL 5000 bp, 1-6: Different samples from clinical case, 7: Positive control and 8: Negative control
